# Two-Stage Liver Transplantation with Temporary Porto-Middle Hepatic Vein Shunt

**DOI:** 10.1155/2010/570392

**Published:** 2010-08-09

**Authors:** Giovanni Varotti, Enzo Andorno, Marco Casaccia, Stefano Di Domenico, Giuliano Bottino, Pietro Diviacco, Nicola Morelli, Chiara Ferrari, Roberto Ferrante, Umberto Valente

**Affiliations:** Department of Surgery and Transplantation, San Martino Hospital, University of Genoa, Monoblocco IV, Largo R. Benzi 10, 16132 Genoa, Italy

## Abstract

Two-stage liver transplantation (LT) has been reported for cases of fulminant liver failure that can lead to toxic hepatic syndrome, or massive hemorrhages resulting in uncontrollable bleeding. Technically, the first stage of the procedure consists of a total hepatectomy with preservation of the recipient's inferior vena cava (IVC), followed by the creation of a temporary end-to-side porto-caval shunt (TPCS). The second stage consists of removing the TPCS and implanting a liver graft when one becomes available. We report a case of a two-stage total hepatectomy and LT in which a temporary end-to-end anastomosis between the portal vein and the middle hepatic vein (TPMHV) was performed as an alternative to the classic end-to-end TPCS. The creation of a TPMHV proved technically feasible and showed some advantages compared to the standard TPCS. In cases in which a two-stage LT with side-to-side caval reconstruction is utilized, TPMHV can be considered as a safe and effective alternative to standard TPCS.

## 1. Introduction

The two-stage total hepatectomy with temporary portocaval shunt and subsequent liver transplantation (LT) were first described by Ringe et al. [1] in 1988. The rationale for the procedure is based upon the concept that patients with fulminant hepatic failure or graft failure that can lead to a toxic hepatic syndrome, or massive haemorrhages resulting in uncontrollable bleeding, can benefit from urgent removal of the native liver followed by LT when an organ becomes available [2, 3].

The first stage of the procedure consists of a total hepatectomy with preservation of the recipient's inferior vena cava (IVC) and suture of the three hepatic veins. Then a temporary end-to-side portocaval shunt (TPCS) is created to re-establish the splanchnic outflow during the anhepatic phase. The second stage consists of removing the TPCS and implanting the liver graft when one becomes available [4].

We report a case of a two-stage total hepatectomy and LT in which a temporary end-to-end anastomosis between the portal vein and the middle hepatic vein (TPMHV) was performed as an alternative to the classic end-to-end TPCS.

## 2. Case Report

A 43-year-old woman underwent urgent laparotomy for spontaneous massive rupture of a subcapsular liver hematoma associated with HELLP syndrome.

The right lobe of the liver was entirely replaced by a large hematoma and areas of necrosis, and there were deep ruptures in both lobes. After establishing that neither conservative surgical treatments nor partial resections would have been effective; a total hepatectomy was performed to control the hemorrhage.

**Figure 1:**
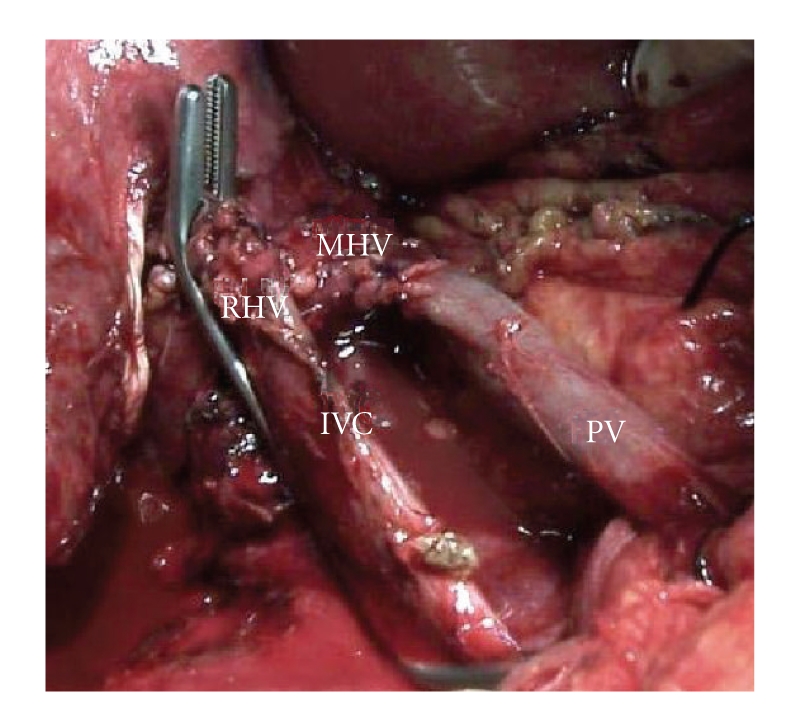
Anhepatic phase: the IVC is tangentially clamped but the portal flow is guaranteed by the TPMHV which lies just above the caval clamp (IVC: inferior vena cava, MHV: middle hepatic vein shunt, RHV: right hepatic vein, and PV: portal vein).

After isolating the hilar structures, the bile duct and hepatic artery were ligated and divided. The portal vein (PV) was clamped and divided at the bifurcation level. After division of all ligamentous attachments, the liver was progressively mobilized to the left until the retrohepatic vena cava was completely exposed. All the accessory hepatic veins encountered were ligated and divided, and the right hepatic vein was clamped, divided, and oversewn. Rotating the liver towards the right, the left and middle hepatic veins were isolated for 3-4 cm within the hepatic parenchyma and divided permitting the removal of the liver. After suturing the stump of the left hepatic vein, it was noted that the orifice of the stump of the middle hepatic vein matched the orifice of the portal vein in length and diameter. A temporary end-to-end porto-middle hepatic vein anastomosis was created using a running suture (Prolene 5/0; Ethicon, Somerville, NJ), instead of the standard TPCS (Figure 1).

The patient was taken back to the ICU for hemodynamic and metabolic monitoring and management. Twelve hours after placing the patient on the liver transplant waiting list with a Status 1 designation, a liver became available. 

**Figure 2:**
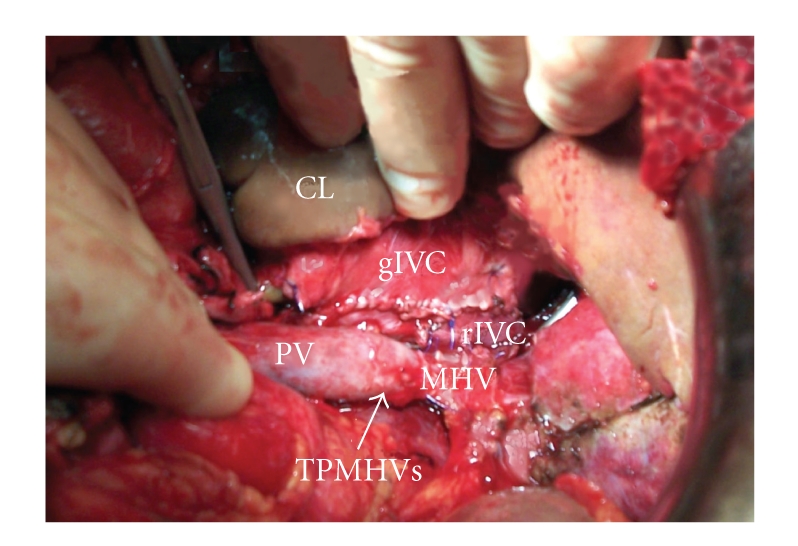
Creation of the end-to-side caval anastomosis is not impeded by the presence of the TPMHV (CL: caudate lobe, rIVC: recipient's inferior vena cava, gIVC: graft's inferior vena cava, MHV: middle hepatic vein, PV: portal vein, and TPMHV: temporary porto-middle hepatic vein shunt).

The patient returned to the operating room for a LT which was performed in a piggyback fashion. The recipient IVC was cross-clamped tangentially, occluding approximately half of its lumen, but leaving the TPMHV open and untouched due to its position above the caval clamp. After a longitudinal incision of the recipient IVC anterior wall, the graft was placed orthotopically and rotated to the right, and an end-to-side anastomosis between donor and recipient IVC was performed (Figure 2).

Next, the TPMHV was taken down, and an end-to-end PV anastomosis was completed. After removing the IVC and portal vein clamps, the liver perfused well, and the patient remained hemodynamically stable.

Arterial and biliary anastomoses were constructed utilizing a standard technique. 

The patient was discharged 47 days after the liver transplant with normal hepatic, renal kidney, and respiratory functions. She remains in healthy condition after ten-month followup.

## 3. Discussion

The creation of a TPMHV as described above proved technically feasible and showed some advantages over the standard TPCS.

Firstly, this technique avoids the partial IVC clamping that is required for the creation of the traditional TPCS which may have a negative impact on a patient who is hemodynamically compromised [5]. In contrast, the creation of the TPMHV requires only the clamping of the origin of the middle hepatic vein alone, leaving the caval flow untouched.

Secondly, during implantation of the graft, the positioning manoeuvre of the caval clamp longitudinally on the IVC and the subsequent creation of a side-to-side caval anastomosis can be made more difficult in the presence of placement of a TPCS which usually lies on the lower portion of the retrohepatic IVC.

The use of a TPMHV permitted the unimpeded placement of the IVC clamp as well as the creation of the caval anastomosis. 

Moreover, throughout the ICV clamping phase, because the standard TPCS is placed upstream of the caval clamp, the portal flow becomes unavoidably at least partially obstructed, with consequent increased risk of splanchnic congestion and hemodynamic alterations. This situation is avoided by the TPMHV which remains downstream of the caval clamp, allowing full portal flow throughout the procedure.

In conclusion, in cases of two-stage LT procedures with side-to-side caval reconstruction, TPMHV can be considered as a safe and effective alternative to standard TPCS.

In addition to this setting, this shunt technique may also be applied in living donor liver transplantation (LDLT). 

A recent report from Kyoto [6] described a series of LDLTs in which the authors performed a hemi-portocaval shunt when the PV pressure was ≥20 mmHg at the time of laparotomy, as prevention of small size syndrome.

To avoid splanchnic congestion during the anhepatic phase, a TPCS between the IVC and PV branch constructed in all cases. In the case of hemi-portocaval shunt, the distal end of the PV vein branch for the shunt was extended with a vein graft in advance and anastomosed to the IVC, since a short shunt may seriously hamper adequate mobilization and anastomosis between the graft PV and the other branch of the recipient PV.

In these cases, using a right lobe graft, we think that a TPMHV between the left branch of the portal vein and the MHV may be a useful alternative to the TPCS in that it would not impede the venous or portal anastomoses, and when necessary, it could be left in place replacing the hemi-portocaval shunt.
